# Effects of different doses of vancomycin powder in total knee and hip arthroplasty on the periprosthetic joint infection rate: a systematic review and meta-analysis

**DOI:** 10.1186/s13018-022-03445-2

**Published:** 2022-12-17

**Authors:** Shiyu Liao, Zhize Yang, Xiao Li, Jintian Chen, Jian-guo Liu

**Affiliations:** Department of Orthopaedics, The First Hospital of Jilin University, No. 71, Xinmin Street, Chaoyang District, Changchun, 130000 Jilin China

**Keywords:** Periprosthetic joint infection, THA, TKA, TJA, Vancomycin

## Abstract

**Background:**

Periprosthetic joint infection (PJI) following total joint arthroplasty (TJA) is a serious complication for patients. Some joint surgeons have tried to use vancomycin powder (VP) in total knee and total hip arthroplasty to prevent postoperative PJI, but its effect is still not clear. At present, there is no meta-analysis that specifically analyses the effect of different doses of vancomycin powder on the incidence of PJI.

**Methods:**

We carried out a search based on the Preferred Reporting Items for Systematic Reviews and Meta-Analyses (PRISMA) guidelines and identified the studies we needed. Review Manager (RevMan) 5.3 software was employed for statistical analysis.

**Results:**

The analysis of primary TKA (PTKA) showed that using 1 g (RR 0.38, 95% CI 0.22–0.67 [*P* = 0.0008]) and 2 g (RR 0.48, 95% CI 0.31–0.74 [*P* = 0.0008]) of vancomycin powder in primary TKA (PTKA) could all significantly prevent PJI. The analysis of primary THA (PTHA) showed that using 1 g (RR 0.37, 95% CI 0.17–0.80 [*P* = 0.01]) of vancomycin powder effectively decreased the incidence of PJI, while using 2 g (RR 1.02, 95% CI 0.53–1.97 [*P* = 0.94]) of vancomycin powder had no significant effect on preventing PJI. Because the data were abnormal, we believed the conclusion that using 2 g of vancomycin powder in primary THA had no effect on preventing PJI was doubtful. Using vancomycin powder in revision TKA (RTKA) significantly reduced the PJI rate (RR 0.33, 95% CI 0.14–0.77 [*P* = 0.01]), similar to revision THA (RTHA) (RR 0.37, 95% CI 0.14–0.96 [*P* = 0.04]).

**Conclusions:**

In primary TKA, both 1 g and 2 g of vancomycin powder can effectively prevent PJI. In primary THA, using 1 g of vancomycin powder is a better choice, while the effect of using 2 g of vancomycin powder is not clear, and a more prospective randomized controlled trial should be done to verify it. In revision TKA and revision THA, vancomycin powder is a good choice to prevent PJI.

**Supplementary Information:**

The online version contains supplementary material available at 10.1186/s13018-022-03445-2.

## Introduction

Periprosthetic joint infection (PJI) following TJA is a serious complication for patients. Patients who experience PJI must undergo debridement, antibiotics and implant retention (DAIR) or revision surgery, and they bear tremendous psychological and economic burdens. Surveys have shown that patients undergoing TJA due to PJI have a poorer functional prognosis and less satisfaction with the surgery than patients undergoing revision for other reasons, which leads to more lasting negative consequences [1]. Endogenous or exogenous bacteria that infect the joint area following surgery may induce PJI [2]. The most common pathogen is *coagulase-negative staphylococci* [3], but infection caused by fungi should not be ignored as extending antibiotic prophylaxis may cause a shift in causative organisms. Surgeons have adopted various methods for preventing infection after surgery [4], but the postoperative PJI rate after THA is still between 0.86% and 1.03% [5] and the postoperative PJI rate after TKA is between 1.41% and 2.01% [6, 7].

To further reduce the infection rate, some surgeons have tried to use vancomycin powder in wounds. Vancomycin is a glycopeptide antibiotic that achieves a bactericidal effect by inhibiting the synthesis of the cell wall of gram-positive bacteria [8]. Vancomycin powder has already been used by spinal surgeons to prevent deep infections after surgery and has a good effect [9, 10]. However, there is no consensus on the effect of vancomycin powder in joint surgery. Some authors think that local vancomycin powder can prevent PJI [11–18], but some authors think that vancomycin powder is not effective in preventing postoperative infections [19–24]. At present, there are few meta-analyses of whether vancomycin powder can reduce the postoperative PJI rate, and there is much controversy.

In the published meta-analyses, the authors mainly compared the PJI rate of total joint arthroplasty without considering the dose of vancomycin powder, and they did not report whether there was any difference in the effects of vancomycin powder at different doses. In addition, the effects of vancomycin powder on the PJI rate of primary arthroplasty and revision arthroplasty may also be different. Therefore, we conducted this study to answer the following questions:

1. Can vancomycin powder effectively prevent PJI after primary TKA? Whether different doses of vancomycin powder can all prevent PJI after primary TKA or not?
2. Can vancomycin powder effectively prevent PJI after primary THA? Whether different doses of vancomycin powder can all prevent PJI after primary THA or not?
3. Can vancomycin powder used in revision TKA and revision THA prevent PJI?

## Data sources and methods

We registered our study in the PROSPERO database on October 24, 2021 (registration number: CRD42021287003) [25], and was performed based on the PRISMA guidelines [26].

### Literature screening

Five researchers searched four major databases (PubMed, Embase, Cochrane Library and WOS) for documents up until 2021–11–10. The criteria for inclusion articles were as follows: (1) studies that reported on the use of intrawound vancomycin powder during primary and revision THA and TKA; (2) follow-up time ≥ 3 months; (3) retrospective and prospective studies; and (4) the language of the study is English. Studies with less than 3-month follow-up, those without complete infection data, non-English studies, and without full-text availability were excluded. The search terms included vancomycin (MeSH); arthroplasty, replacement, knee (MeSH); arthroplasty, replacement, hip (MeSH); total hip replacement (MeSH); and total knee arthroplasty (MeSH) (for the detailed search strategy, see Additional file 1: Table S1).

### Data extraction

After the screening, qualified articles were assessed entirely based on the same inclusion and exclusion criteria and the characteristics extracted from databases included the first author’s name, the year of publication, evidence level, study types, vancomycin powder dosage, different types of surgery, sample size of experimental and control groups, follow-up time, and number of PJI occurrences in the vancomycin-treated group and control group. We used the Newcastle–Ottawa Scale (NOS) [27] to evaluate the quality of studies, with 7–9 points indicating high quality.

### Publication bias

The publication bias is evaluated by a flow diagram, where the x-axis represents the effect size (RR). The smaller the value is, the closer it is to the left end. The vertical line in the middle is the ideal effect value. Ideally, the studies should be evenly distributed on both sides of the vertical line. The y-axis represents the standard error, which is the sample size. The larger the sample size is, the higher the distribution and the smaller the standard error.

### Statistical analysis

The PJI rate of each study was utilized to calculate the relative risk (RR) and confidence intervals (CIs). The chi-square test was used to measure study heterogeneity. If *I*^2^ was between 25 and 50%, the heterogeneity was considered small; if *I*^2^ was between 50 and 75%, the heterogeneity was considered moderate; if *I*^2^ > 75%, the heterogeneity was significant [28, 29]. When *I*^2^ is more than 50%, the random-effects model is employed for the evaluation; alternatively, the fixed-effects model is applied. RevMan 5.3 software was employed to perform all statistical analyses.

## Results

### Search results

**Fig. 1:**
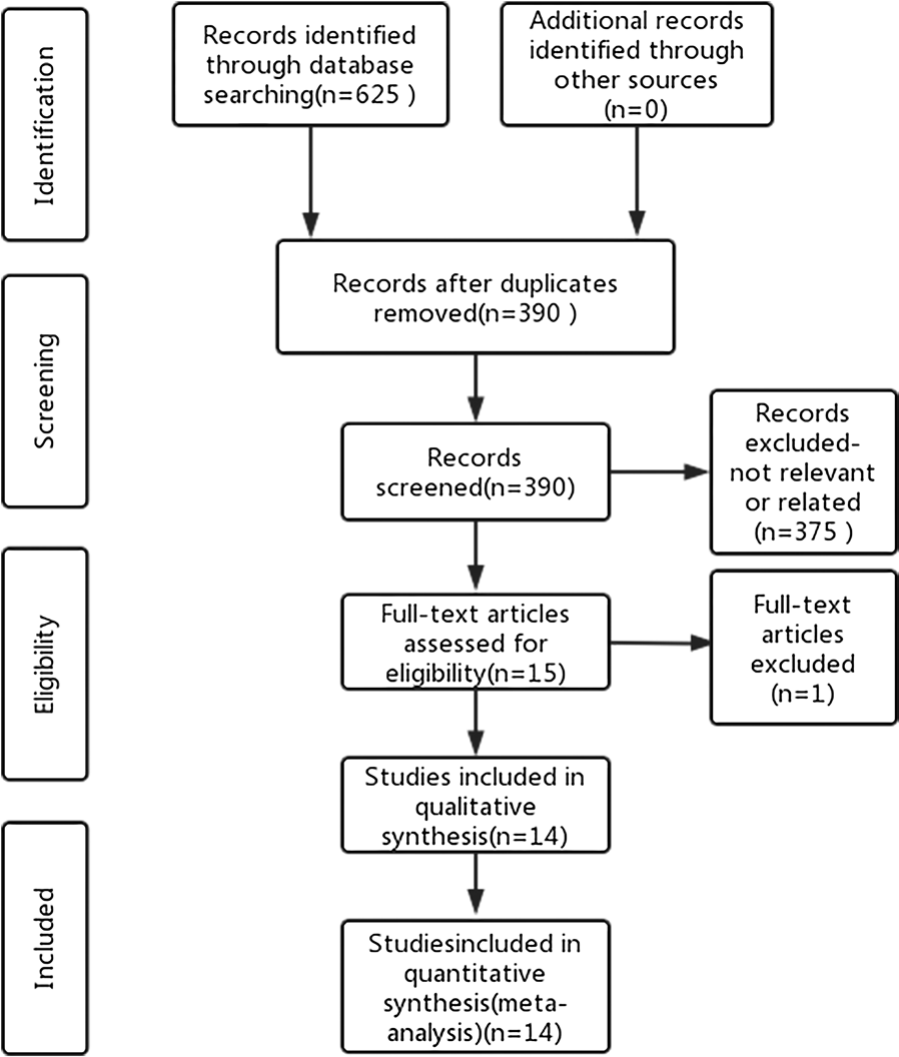
PRISMA flow diagram of the search strategy

**Fig. 2:**
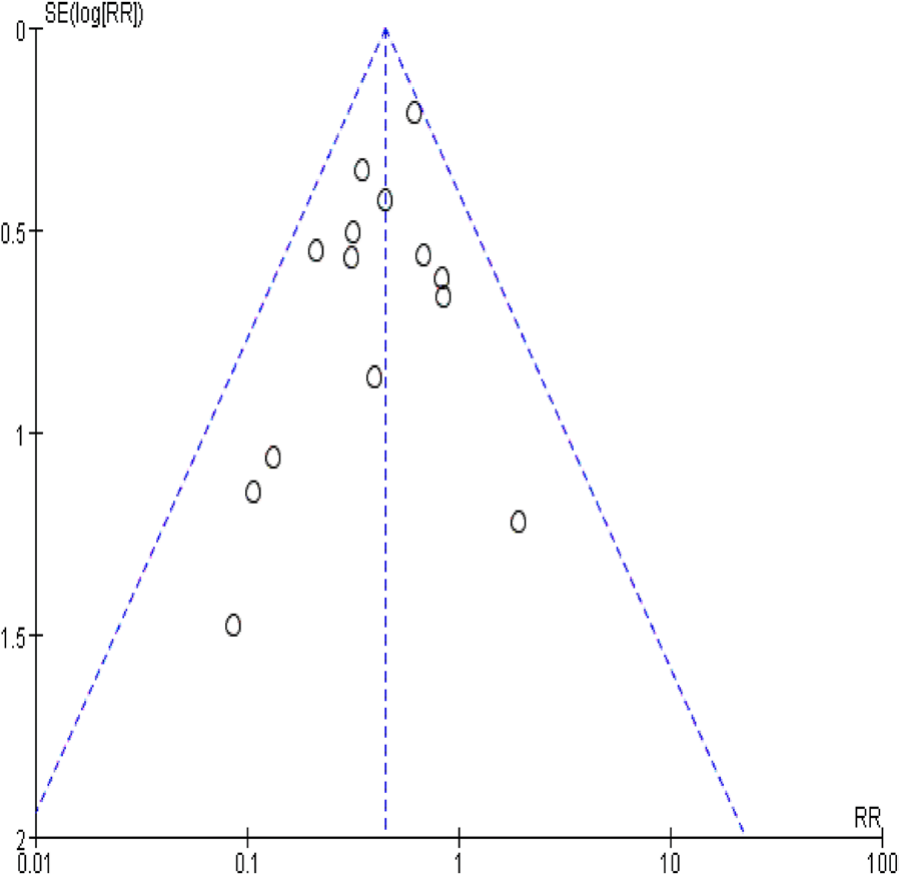
Funnel plot of included studies

The full text of eligible articles was filtered according to inclusion and exclusion criteria, and different articles were reviewed by another author. We excluded duplicate studies and screened studies individually, and then we evaluated the full text of the included studies to exclude studies that did not fulfil the requirements. The screening process is shown in Fig. 1. We searched the articles we needed in the PubMed, Web of Science, Embase and Cochrane Library databases. A total of 625 articles were screened out in the first screening, and 14 original articles were ultimately included. The funnel plot (Fig. 2) showed no indication of publication bias among the 14 studies.

### Research nature

This analysis comprised 14 studies (Additional file 2: Table S2), 12 of which were retrospective cohort studies and 2 of which were prospective cohort studies. The study comprised 35,418 participants, including 20,368 treated with vancomycin powder and 15,050 not treated with vancomycin powder. Based on the NOS (Table 1), all studies scored at least 7 points and indicated that they were all high-quality studies.

### Statistical results

#### Can vancomycin powder effectively prevent PJI after primary TKA? Whether different doses of vancomycin powder can all prevent PJI after primary TKA or not?

**Fig. 3:**
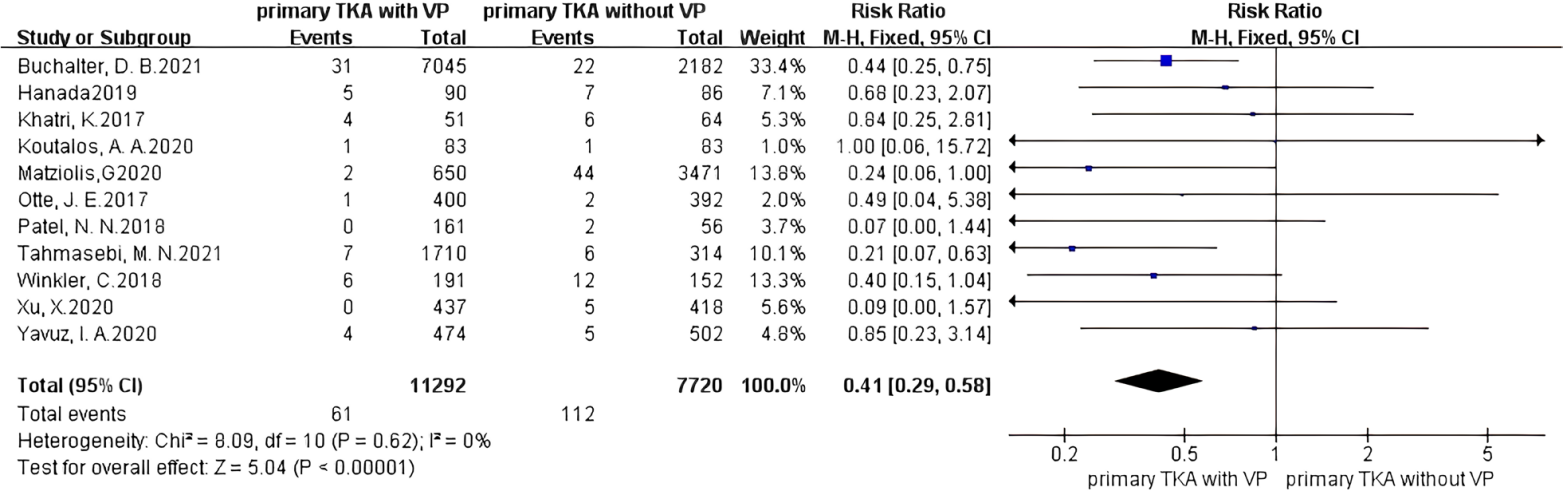
Forest plot of the risk of PJI after PTKA with VP

**Fig. 4:**
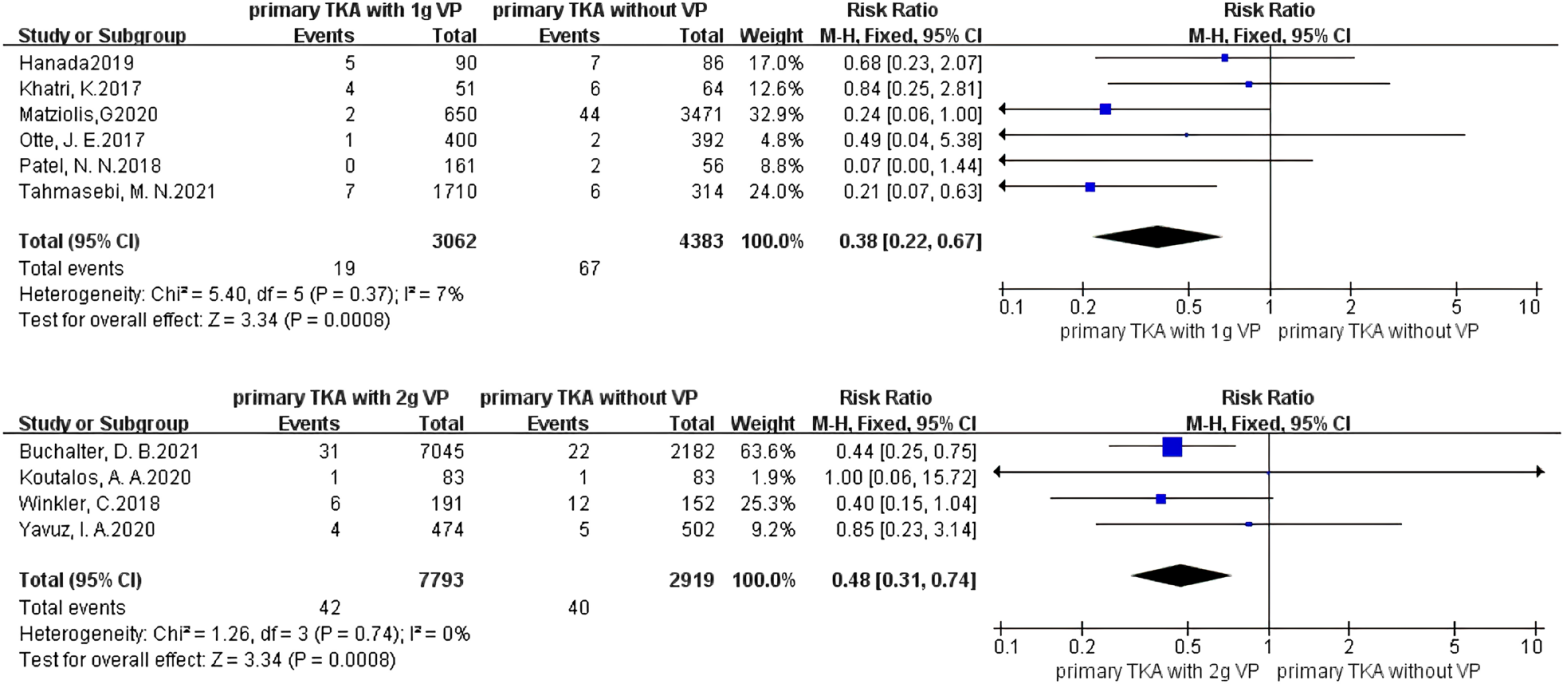
Forest plot of the risk of PJI after PTKA with different doses of VP

The analysis of the primary TKA (PTKA) showed that the incidence of PJI was significantly reduced when using vancomycin powder (Fig. 3), with no heterogeneity existing in the studies (*I*^2^ = 0%, *P* = 0.62), and PJI was significantly prevented in patients treated with vancomycin powder (RR 0.41, 95% CI 0.29–0.58 [*P* < 0.00001]). For 1 g of vancomycin powder (Fig. 4), no heterogeneity existed in the studies (*I*^2^ = 7%, *P* = 0.37), and the effect on preventing PJI in the vancomycin-treated group was significantly different from that in the control group (RR 0.38, 95% CI 0.22–0.67 [*P* = 0.0008]). For 2 g of vancomycin powder (Fig. 4), no heterogeneity existed in the studies (*I*^2^ = 0%, *P* = 0.74), and the effect on preventing PJI in the vancomycin-treated group was significantly different from that in the control group (RR 0.48, 95% CI 0.31–0.74 [*P* = 0.0008]). It is suggested that both the use of 1 g and 2 g of vancomycin powder in primary TKA can significantly reduce the PJI rate.

#### Can vancomycin powder effectively prevent PJI after primary THA? Whether different doses of vancomycin powder can all prevent PJI after primary THA or not?

**Fig. 5:**
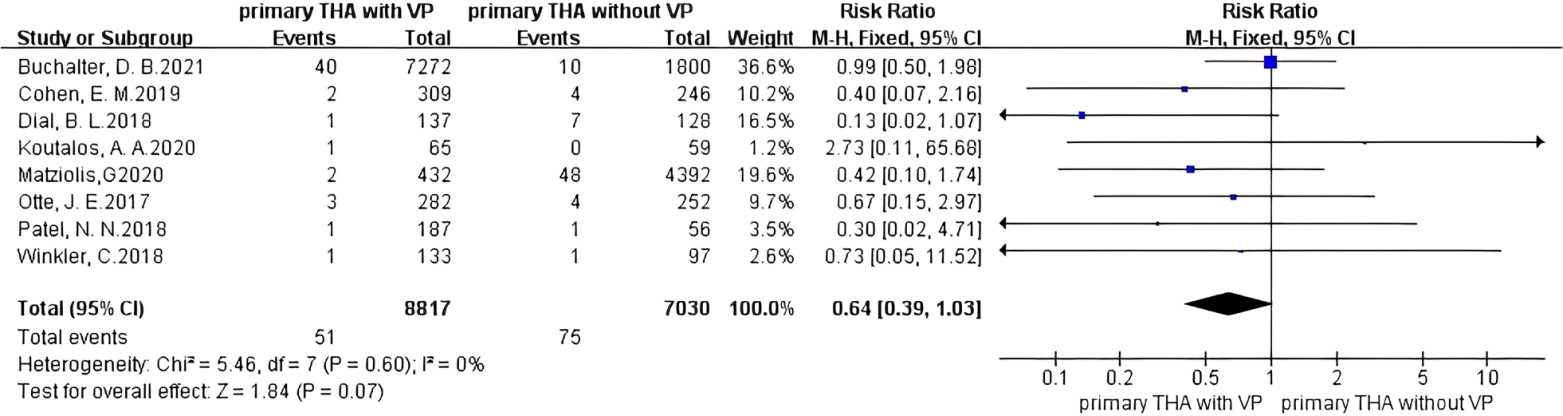
Forest plot of the risk of PJI after PTHA with VP

**Fig. 6:**
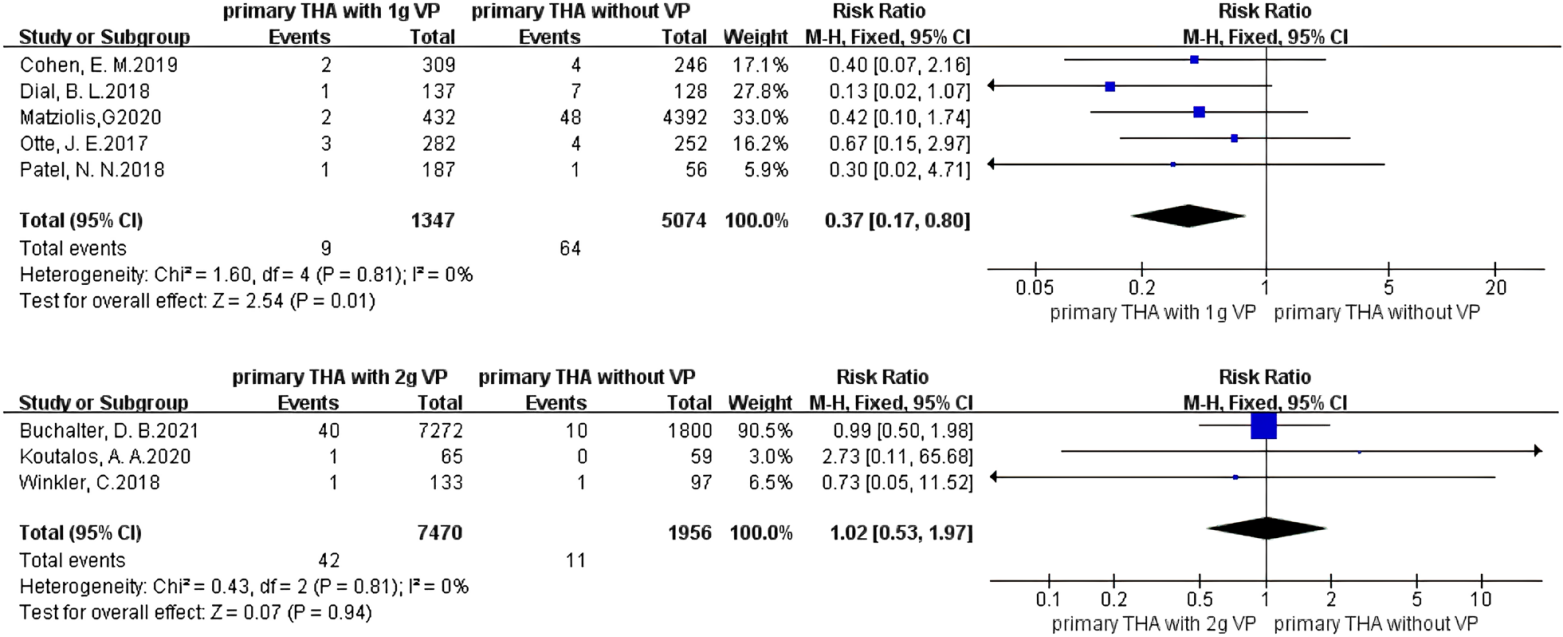
Forest plot of the risk of PJI after PTHA with different doses of VP

An analysis of primary THA (PTHA) showed that the incidence of PJI was not significantly reduced when using vancomycin powder (Fig. 5), and no heterogeneity existed in the studies (*I*^2^ = 0%, *P* = 0.60). Vancomycin powder had no obvious preventive effect on PJI (RR 0.64, 95% *CI* 0.39–1.03 [*P* = 0.07]), but the relative risk ratio was 0.64, indicating that the incidence of PJI in the vancomycin-treated group had a decreasing trend. For 1 g of vancomycin powder (Fig. 6), no heterogeneity existed in the studies (*I*^2^ = 0%, *P* = 0.81), and the PJI rate was significantly decreased in patients treated with vancomycin powder (RR 0.37, 95% CI 0.17–0.80 [*P* = 0.01]). For 2 g of vancomycin powder (Fig. 6), no heterogeneity existed in the studies (*I*^2^ = 0%, *P* = 0.81), and PJI was not effectively prevented in the vancomycin-treated group (RR 1.02, 95% CI 0.53–1.97 [*P* = 0.94]). It is suggested that in primary THA, using 1 g of vancomycin powder had a significant effect on reducing the PJI rate, while the effect was not obvious when 2 g of vancomycin powder was used.

#### Can vancomycin powder used in revision TKA and revision THA prevent PJI?

**Fig. 7:**
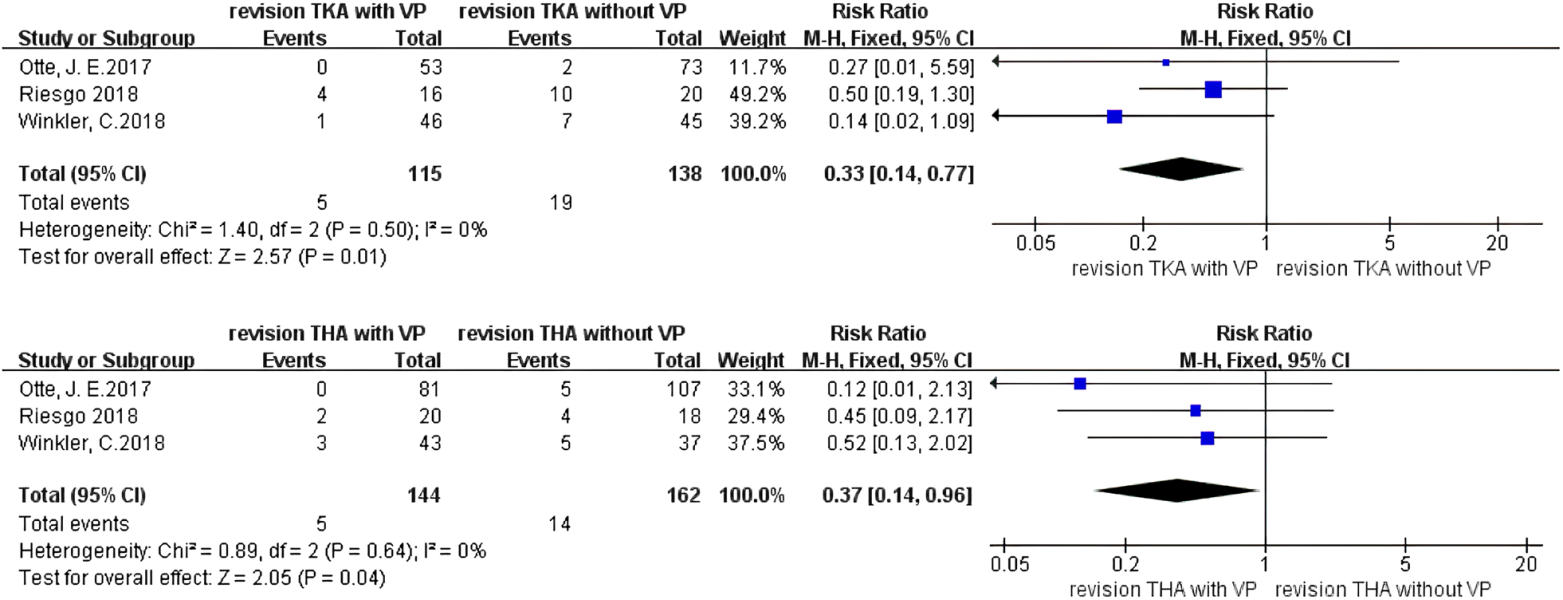
Forest plot of the risk of PJI after RTKA and RTHA with VP

A separate analysis of revision TKA (RTKA) (Fig. 7) showed that the incidence of PJI was significantly decreased in the vancomycin-treated group (RR 0.33, 95% CI 0.14–0.77 [*P* = 0.01]), and no heterogeneity existed in the studies (*I*^2^ = 0%, *P* = 0.50). This suggests that using vancomycin powder in revision TKA can significantly decrease the incidence of PJI.

A separate analysis of revision THA (RTHA) (Fig. 7) showed that the incidence of PJI was significantly decreased in the vancomycin-treated group (RR 0.37, 95% CI 0.14–0.96 [*P* = 0.04]), and no heterogeneity existed in the studies (*I*^2^ = 0%, *P* = 0.64). This suggests that using vancomycin powder in revision THA can significantly decrease the incidence of PJI.

## Discussion

Vancomycin powder was first used in spinal surgery to prevent deep postoperative infections. Infection of the surgical site after spinal surgery is a serious complication [30]. According to the guidelines of the North American Spine Association [31], cefazolin is usually used in spinal surgery to prevent infections caused by *Staphylococcus aureus*. However, the number of cases of infections with methicillin-resistant *Staphylococcus aureus* (MRSA) is increasing [32–35]. Based on data from the USA and the UK [32–35], approximately 50% of infected patients in the ICU have MRSA infections. Therefore, vancomycin powder began to be used clinically. In the literature on spinal surgery, the infection rate significantly decreased after using vancomycin powder in lumbar spine surgery [36], cervical spine surgery [37], and some deformity surgeries [38].

Vancomycin powder in joint arthroplasty surgery has been used only in recent years, and the effect of reducing the postoperative PJI rate is still unclear. According to the results of some previously published meta-analyses [39–41], the use of vancomycin powder has a significant effect on reducing the postoperative PJI rate. However, we believe that the published meta-analyses are not comprehensive and have not considered the effects of different surgical sites, different surgery types, and different vancomycin powder doses on the PJI rate.

Therefore, we performed this meta-analysis to analyse the effect of different doses of vancomycin powder in preventing PJI in joint arthroplasty. In the analysis of primary TKA in this study, we found that 1 g or 2 g of vancomycin powder could all prevent PJI. In the analysis of primary THA, we found that 1 g of vancomycin powder reduced the incidence of PJI. However, no significant change in the incidence of PJI was found in the experimental group when 2 g of vancomycin powder was used in primary THA. This result might be contrary to clinical experience. Overall, in the study of using 1 g of vancomycin powder, the PJI rates of the experimental group and control group were 0.67% and 1.26%, respectively; in the study of using 2 g of vancomycin powder, the PJI rates of the experimental group and control group were all 0.56%. We analysed the data and found that the PJI rate of experimental group treated with 2 g of vancomycin powder (0.56%) was slightly lower than that treated with 1 g of vancomycin powder (0.67%), and this result was logical. However, the PJI rate of the control group in the study of using 2 g of vancomycin powder (0.56%) was much lower than that in the study of using 1 g of vancomycin powder (1.26%). These data were obviously abnormal and there might be a potential bias that was not detected by the classical tools of systematic reviews. In addition, there were only 1,956 patients included in the control group of the study of using 2 g of vancomycin powder (compared to the study of using 1 g of vancomycin powder, 5074 patients were included in the control group). The small number of patients enrolled might also affect the accuracy of the results. So we believed the conclusion that using 2 g of vancomycin powder in primary THA had no effect on preventing PJI was doubtful, and more prospective randomized controlled trial studies were needed to verify it. Furthermore, the failure to include more studies on using 2 g of vancomycin powder in primary THA was also one of the disadvantages of this paper.

Part of the studies included in this paper had a follow-up period of only 3 months. However, a large proportion of PJI occurred more than 1 year after surgery. If the follow-up time is too short, some PJI may not be detected so that affecting the accuracy of the results. However, we believe that the PJI that occurred more than 1 year after surgery is more related to host factors, and less related to the surgical technique of the surgeon and the treatment regimen of vancomycin powder. Therefore, we think that the follow-up period of 3 months has a limited influence on the accuracy of the results.

The advantages of this study are as follows: (1) Most of the previously published meta-analyses on whether vancomycin powder can prevent PJI following joint arthroplasty did not separate the comparisons for TKA and THA or those for primary arthroplasty and revision arthroplasty. (2) To the best of our knowledge, there is no specific meta-analysis that analyses the effect of different doses of vancomycin powder. This study not only analysed the different surgical sites and different surgical types but also studied the effect of vancomycin powder at different doses on PJI, making the research more comprehensive. The disadvantages of this study are as follows: (1) There are few literature reports on the effect of vancomycin powder on the PJI rate in revision joint arthroplasty, and there is no specific analysis of the effect of different doses of vancomycin powder. (2) In view of the result that the use of 2 g of vancomycin powder in THA has no obvious effect on the prevention of PJI, we made a corresponding conjecture, but there is no specific experiment to support this. (3) The failure to include more studies on using 2 g of vancomycin powder in primary THA might influence the accuracy of the final results.

## Conclusion

These results suggest that using vancomycin powder in primary TKA has a rather clear effect on preventing PJI. Both 1 g and 2 g of vancomycin powder can be used for surgery. For primary THA, it is better to use 1 g of vancomycin powder for treatment. The effect of 2 g of vancomycin powder on preventing PJI is not clear, and more prospective randomized controlled trial studies are needed to verify it. Using vancomycin powder in revision TKA and revision THA to reduce the incidence of PJI is relatively effective, but the preventive effect of different doses of vancomycin powder on PJI in revision TKA and THA needs to be verified by more studies.

**Table 1:** Quality assessment according to the NOS of each cohort study

Study	Selection	Comparability	Outcome	Total score
Buchalter, D. B	3	2	3	8
Cohen, E. M	4	2	3	9
Dial, B. L	3	2	3	8
Khatri, K	3	2	3	8
Koutalos, A. A	4	2	3	9
Otte, J. E	3	2	2	7
Patel, N. N	3	2	3	8
Tahmasebi, M. N	3	2	3	8
Winkler, C	3	2	2	7
Xu, X	3	2	3	8
Yavuz, I. A	3	2	3	8
Riesgo	3	2	3	8
Hanada	3	2	3	8
Matziolis,G	3	2	3	8

## Supplementary Information

**Additional file 1**. **Table S1.** Search strategy.

**Additional file 2**. **Table S2.** Characteristics of patients extracted from all included studies.

## Data Availability

All data generated or analysed during this study are included in this published article [and its supplementary information files].

## Abbreviations

PJI: Periprosthetic joint infection
TJA: Total joint arthroplasty
TKA: Total knee arthroplasty
THA: Total hip arthroplasty
PTKA: Primary total knee arthroplasty
PTHA: Primary total hip arthroplasty
RTKA: Revision total knee arthroplasty
RTHA: Revision total hip arthroplasty
PRISMA: Preferred Reporting Items for Systematic Reviews and Meta-Analyses
VP: Vancomycin powder
NOS: Newcastle–Ottawa Scale
